# Tree cover and species composition effects on academic performance of primary school students

**DOI:** 10.1371/journal.pone.0193254

**Published:** 2018-02-23

**Authors:** Sivajanani Sivarajah, Sandy M. Smith, Sean C. Thomas

**Affiliations:** Faculty of Forestry, University of Toronto, Toronto, Ontario, Canada; Charles P. Darby Children's Research Institute, UNITED STATES

## Abstract

Human exposure to green space and vegetation is widely recognized to result in physical and mental health benefits; however, to date, the specific effects of tree cover, diversity, and species composition on student academic performance have not been investigated. We compiled standardized performance scores in Grades 3 and 6 for the collective student body in 387 schools across the Toronto District School Board (TDSB), and examined variation in relation to tree cover, tree diversity, and tree species composition based on comprehensive inventories of trees on school properties combined with aerial-photo-based assessments of tree cover. Analyses accounted for variation due to socioeconomic factors using the learning opportunity index (LOI), a regional composite index of external challenges to learning that incorporates income and other factors, such as students with English as a second language. As expected, LOI had the greatest influence on student academic performance; however, the proportion of tree cover, as distinct from other types of “green space” such as grass, was found to be a significant positive predictor of student performance, accounting for 13% of the variance explained in a statistical model predicting mean student performance assessments. The effects of tree cover and species composition were most pronounced in schools that showed the highest level of external challenges, suggesting the importance of urban forestry investments in these schools.

## Introduction

Urban green spaces, specifically urban forests, are important because they moderate air temperature [1], mitigate ambient air pollution [2], produce human health benefits [3–7], lower human mortality rates [8], and generally improve the quality of life of urban inhabitants [9–10]. Human exposure to green space can result in positive feelings, relaxation, and stress relief, and can restore attention-demanding cognitive performance [11–12]. Mental health benefits following exposure to forested areas have also been linked with specific physiological responses, including reduced diastolic blood pressure and reduced heart rate [6–8].

Several studies have examined the relationship of green space in preschool and elementary school playgrounds to desired educational outcomes. These studies consistently show a positive relationship between natural playscapes and enhanced physical activity [13], motor development [14], creative play behavior [13; 15–16], and environmental learning [16]. When exposed to green space, children aged from 7 to 12 years old with attention deficit disorder (ADD) functioned more effectively as their ADD symptoms decreased [11]. In general, these benefits suggest that green space creates a supportive environment for children that may enhance academic performance.

Many factors are known to strongly influence the academic performance of primary and secondary school children, including gender [17–18], ethnicity [17], parental education, occupation, income, and services used by individuals [19–20]. It is expected that these socioeconomic factors generally have a predominant effect on academic performance, and thus must be taken into account in the evaluation of environmental influences on learning outcomes. Two recent studies present evidence for detectable effects of green space on learning outcomes correcting for socioeconomic factors. In a study of secondary schools, Matsuoka [21] found that the amount of green space visible from cafeteria and classroom windows was positively linked with test scores, graduation rates, and percentage of students planning to attend post-secondary education [21]. In a regional study of primary schools, Wu et al. [22] showed a significant positive association between school greenness and academic performance of its students based on remote sensing data. These data thus do not distinguish potential effects of tree cover from other green spaces. To date, no research has utilized tree inventory information to specifically explore the potential influence of tree cover, diversity, or species composition on the academic performance of primary or secondary school children.

In the present study, we make use of comprehensive surveys of trees on school property conducted in collaboration with the Toronto District School Board, in conjunction with temporally matched data on academic performance and socioeconomic factors, to examine potential effects of tree cover, diversity, and species composition on academic performance. We address the following specific questions: (i) How important is schoolyard tree cover on the academic performance of primary school students? (ii) Does tree diversity, independent of tree cover, influence academic performance? (iii) Since most conifers retain leaves through the winter, are effects of conifers, independent of total tree abundance effects, detectable? (iv) Are schoolyard tree effects more pronounced in schools characterized by high socioeconomic limitations to academic performance?

## Methods

### School-level academic and socio-economic data

Data were collected from 387 elementary schools from the Toronto District School Board (TDSB) in Toronto, Ontario (43.7° N, 79.4° W), the most populous city in Canada. An independent agency of the provincial government (Education Quality and Accountability Office (EQAO)) provided the data to measure collective student performance for each school based on the percentage of students at or above the provincial standard in Grade 3 and Grade 6 for reading, writing, and mathematics between the years of 2006 to 2010 (Refer to EQAO, http://www.eqao.com/ for more information). Alternative schools and schools with incomplete EQAO data over the period in question were omitted from analyses.

TDSB’s learning opportunity index (LOI), which evaluates schools based on measures of external challenges affecting student’s academic performance, was also determined for schools in the dataset. LOI is a composite measure based on multiple variables, including median income, percentage of families whose income is below the low-income measure (before taxes), percentage of families receiving social assistance, adults with low education, and adults with university degrees and lone-parent families, compiled into a single index that uses a scale from zero to one (with zero being the lowest level of external challenge and one being the highest) [23]. In addition, socio-demographic and economic data were collected on the neighbourhood of each school, including population, the number of children, average household income, educational level of adults, and proportion of visible minorities by Well-Being Toronto [24]. Schools were treated as the unit of analysis to avoid identifying individual students and to provide an aggregated mean of all variables for each school.

### School-level geospatial and tree inventory data

Using ArcGIS v. 10.2, the following variables were measured for each school: total land area (m^2^), total soft surface (non-treed area (i.e. grasses and shrubs) available for vegetation (m^2^)), tree canopy cover (m^2^), and the ratio of tree canopy cover area to ground area available for vegetation (expressed as % tree cover). For each school, calculations for the land and vegetation were derived from city-wide raster data developed in 2007 and available as part of the Urban Tree Canopy (UTC) Assessment for Toronto [25]. Land and vegetation raster data were converted into polygons and overlaid with school boundaries to calculate the proportion of tree cover, proportion of soft surface, and building area in relation to each school ground. In addition, the number of tree species, conifers, and hardwood trees were determined for each school by using the TDSB’s NeighbourWoods tree inventory data collected in collaboration with the Faculty of Forestry, University of Toronto. The TDSB’s NeighbourWoods is a comprehensive tree database obtained during the years of 2004–2015, and includes information on tree location, site characteristics, species, tree size (i.e. diameter at breast height (cm), tree height (m), and crown width (m)), tree condition (i.e. tree lean, poor branch attachment, stem rot, tree defoliation, crown form) and conflicts between trees and other infrastructure. A total of 20,639 trees are included in the inventory. Using the species information from the tree inventory data, diversity measures, including Fisher’s alpha and the Shannon-Wiener index [26], were calculated for each school.

### Statistical analysis

All statistical analyses were performed using R v.3.12 (R Foundation for Statistical, Vienna, Austria). Multiple regressions of mean school-based results for reading, writing, and mathematics in both Grade 3 (n = 251) and 6 (n = 281) from 2005 to 2010 were carried out using LOI 2009, the proportion of tree cover, land cover data (i.e., soft surface, hard surface) and several diversity measures.

The influence of tree cover was analyzed using Generalized Linear Models (GLMs) for the parameters; proportion of tree cover, proportion of soft surface (available area for vegetation) species diversity indices, proportion of hardwood trees, and proportion of conifer trees against school-based Grade 3 and Grade 6 scores. Socio-demographic and economic factors were controlled during this analysis using LOI 2009. Preliminary analyses showed the spatial autocorrelation of model residuals was low, so spatial effects were not considered in statistical analyses. To examine relative responses of schools relative to socioeconomic factors, schools were separated into two categories based on their LOI score: high (LOI ≥ 5) and low (LOI < 5).

To explore potential effects of tree species composition on academic performance metrics, correspondence analysis (CA) was conducted for the species composition dataset based on proportional stem count data. Species composition data were also incorporated using the proportion of conifers, and the proportions of the commonest species in the data set *(i*.*e*. *Acer platanoides*, *Thuja occidentalis*, *Fraxinus pennsylvanica)* as predictors.

## Results

**Fig 1:**
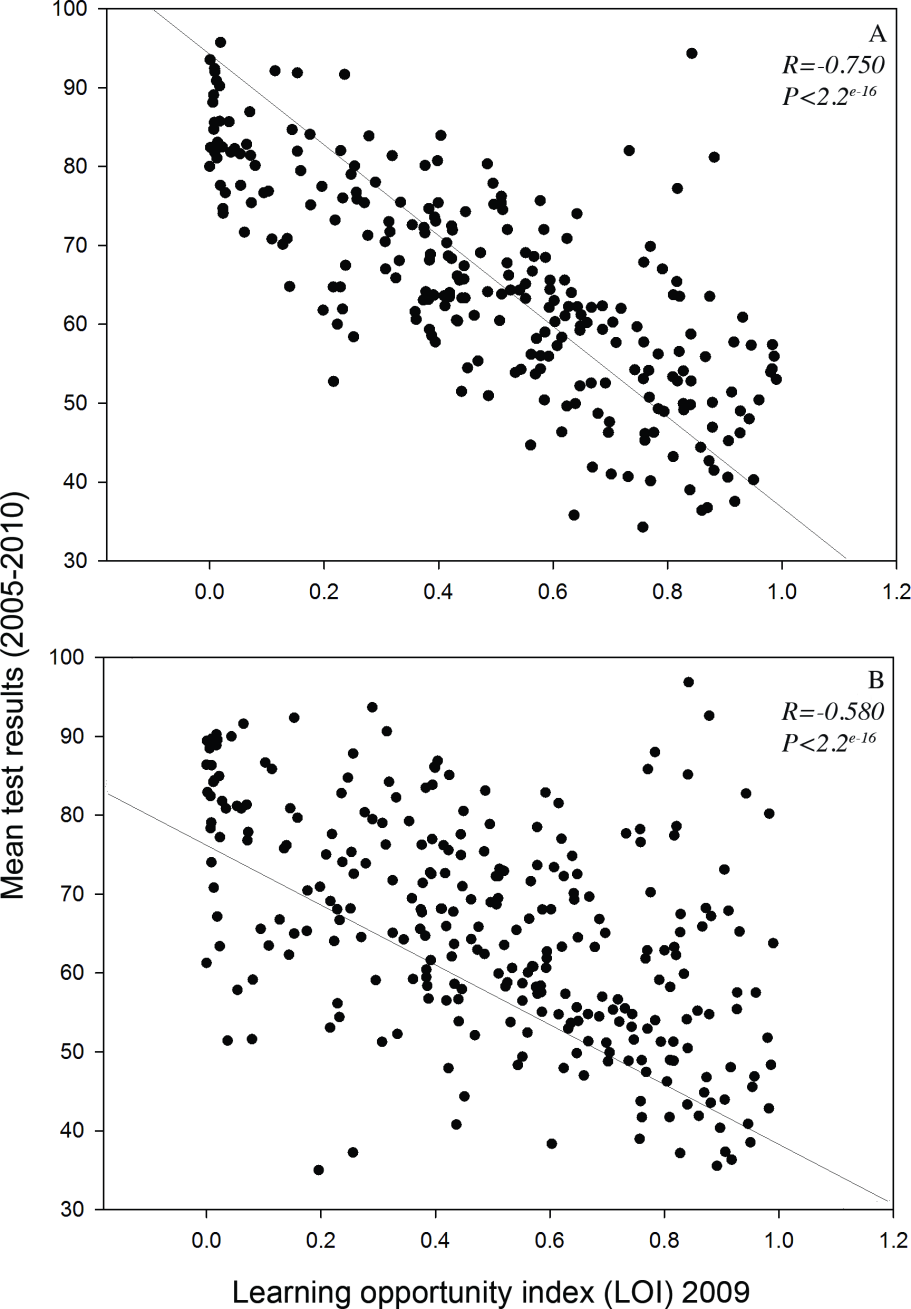
Mean test results per school (percentage of students at or above the provincial standard in 2009) for reading, writing, and math scores in (A) Grade 3 (R = -0.750, P<2.2^e-16^, n = 251) and (B) Grade 6 (R = -0.508, P<2.2^e-16^, n = 281) as a function of a regional aggregated measure of socio-economic constants to learning (the learning opportunity index: LOI), within the Toronto District School Board in Toronto, Ontario, Canada.

**Fig 2:**
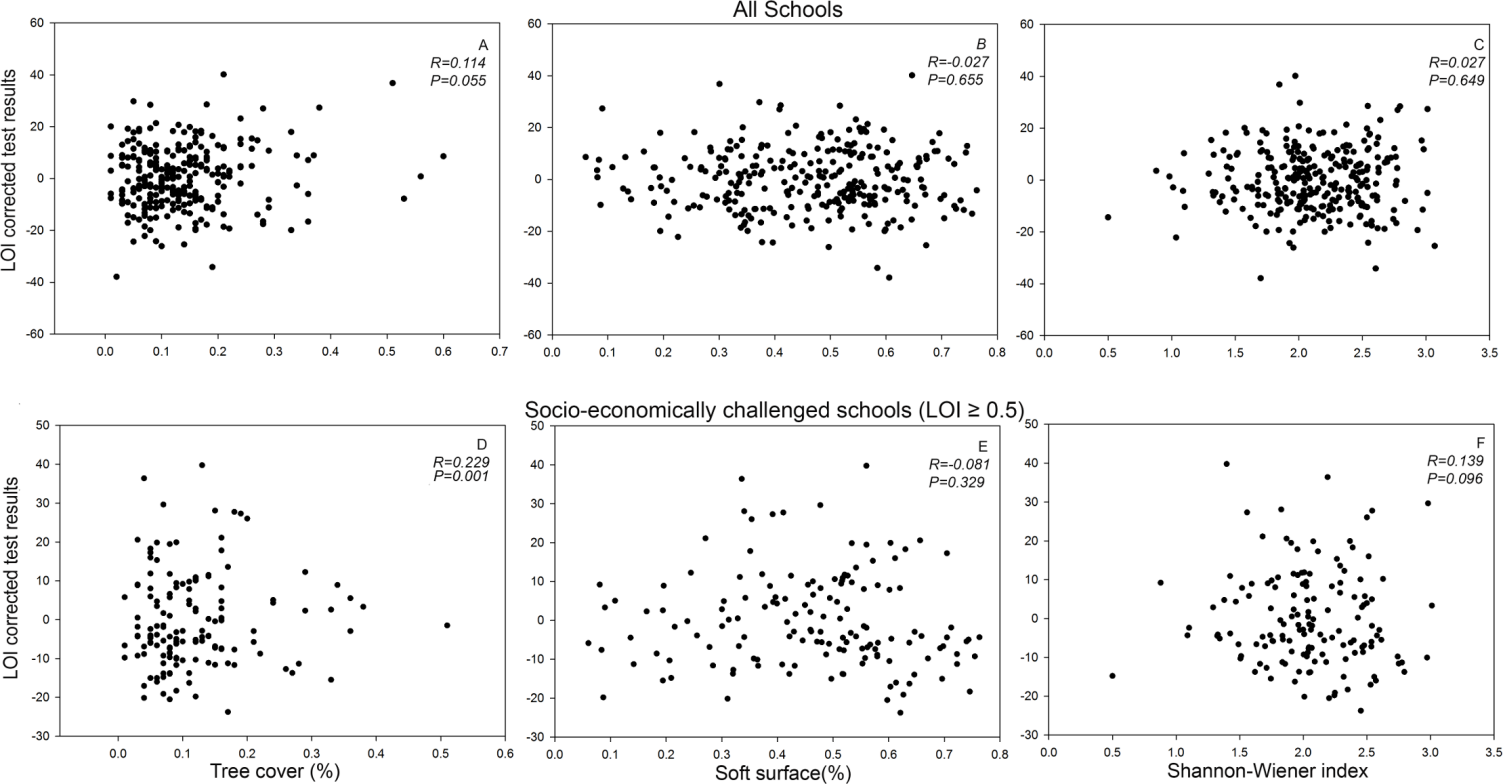
The average test scores of model outputs for Grade 6 students corrected for socio-economic factors using learning opportunity index (LOI) 2009 from all schools (n = 281) compared to (A) tree cover (R = 0.114, P < 0.055) (B) soft surface (R = -0.027, P = 0.655) (C) tree species diversity measured in Shannon-Wiener index (R = 0.028, P = 0.649) and for schools ranked higher on LOI 2009 (LOI ≥ 0.5) (n = 144) (D) tree cover (R = 0.229, P = 0.001) (E) soft surface (R = -0.081, P = 0.329) and (F) tree species diversity based on the Shannon-Wiener index (R = 0.139, P = 0.096). Data are for the Toronto District School Board (TDSB) in Toronto, Ontario, Canada for the years 2005–2010.

Both grade 3 and 6 test results showed a strong positive correlation with LOI scores (R = -0.74, P < 0.001; R = -0.50, P < 0.001) (Fig 1). Tree cover, measured as a proportion of total land area (m^2^), ranged from 1 to 60% among sampled schools, with a mean of 13.6 ± 0.53%(SE); mean test results ranged from 34.3 to 95.7% (grade 3) and 35 to 96.8% (grade 6) among the sampled schools, with a mean of 64.9 ± 0.85% (grade 3) and 65.3±0.83% (grade 6). The additional socio-economic variables from Well-being Toronto were used in preliminary analyses, but did not explain additional variation in EQAO scores. Multiple regressions for test results as a function of LOI and tree cover indicated no significant tree cover influence on grade 3 scores (P = 0.98). However, tree cover effects were detected in grade 6 scores, accounting for 10.7–17.6% (13.0% for mean test scores) of the variance explained by the minimum AICc models predicting student performance assessments (Table 1). Tree cover positively correlated with writing (P = 0.033), whereas reading (P = 0.082), math (P = 0.080), and mean of all test results (P = 0.050) (Fig 2A), were marginally significant (Table 1, Model 2). A linear model was constructed that included a LOI index by tree cover interaction term, and which was found to be significant for total mean test results (P = 0.027), and for test components (reading: P = 0.017; writing: P = 0.029; and math: P = 0.064) (Table 1, Model 3), suggesting the effect of tree cover is different on socio-economically challenged schools than schools ranked lower on the LOI index. To further explore this pattern, schools were separated by LOI scores into highly challenged (LOI ≥ 0.5) and less challenged categories (LOI < 0.5). Tree cover positively correlated with all highly challenged (LOI ≥ 0.5) Grade 6 scores: reading (P = 0.006), writing (P = 0.020), math (P = 0.020), and mean of all test results (P = 0.005) (Table 2, Model 1, Fig 2D). Tree cover was not significant (P = 0.89) as a predictor of test scores for schools experiencing less external challenges (LOI < 0.5). Tree species diversity and proportion of soft surface did not significantly affect children’s test results in all schools (Fig 2B and 2C), regardless of whether data were separated school LOI (Fig 2E and 2F). Similar patterns emerged when the proportion of conifer (%) was added as a predictor in model 3 (Table 1): this variable did not significantly (P = 0.13, P = 0.15) affect children’s test results in all schools or in higher-ranked schools on LOI (≥ 0.5).

**Table 1:** Coefficients and AICc values for alternative generalized linear models (GLMs) are shown as predictors of children’s academic performance quantified as the percent of grade 6 students passing above provincial standards in Toronto, Ontario (n = 281).

Category	Parameters	Model			Model 2			Model 3			
		*B*	P value	AICc	*B*	P value	AICc	*B*	P value	AICc	R^2^
Reading	LOI2009	-25.71	< 0.0001	2221.3	-24.80	< 0.0001	2220.4	-34.60	<0.0001	2216.8	0.253
	Tree cover	_	_		14.38	0.0884		-14.78	0.3205		0.023
	LOI2009xTree cover	_	_		_	_		63.07	0.0183		0.015
Writing	LOI2009	-19.58	<0.0001	2175.2	-18.54	<0.0001	2172.8	-26.77	<0.0001	2170.2	0.187
	Tree cover	_	_		16.25	0.0364		-8.24	0.5472		0.027
	LOIxTree cover	_	_		_	_		52.96	0.0313		0.013
Math	LOI2009	-28.98	< 0.0001	2301.0	-27.88	< 0.0001	2300.0	-36.90	<0.0001	2298.5	0.225
	Tree cover	_	_		17.27	0.0798		-9.55	0.5840		0.018
	LOI2009xTree cover	_	_		_	_		58.01	0.0638		0.009
Mean of all test results	LOI2009	-25.04	<0.0001	2195.8	-24.02	< 0.0001	2194.0	-32.913	<0.0001	2191.1	0.240
	Tree cover	_	_		15.95	0.0509		-10.50	0.4662		0.023
	LOI2009xTree cover	_	_		_	_		57.21	0.0271		0.013

Partial R^2^ values for model terms are listed for Model 3, which shows the minimum AICc values in each case.

**Table 2:** Coefficients and AICc values for alternative generalized linear models (GLMs) are shown as predictors of children’s academic performance quantified as the percent of grade 6 students passing above provincial standards in Toronto, Ontario in schools ranked higher on LOI (≥0.5) (n = 144). CA1 is correspondence analysis on axis 1, and CA2 is correspondence analysis on axis 2 using species composition data.

Category	Parameters	Model 1			Model 2			Model 3		
		B	P value	AICc	B	P value	AICc	B	P value	AICc
Reading	LOI2009	-42.49	0.0014	1128.5	-42.85	0.0013	1129.7	-42.69	0.0014	1131.4
	Tree cover	-77.91	0.2490		-79.40	0.2403		-81.75	0.2281	
	LOI2009xTree cover	147.77	0.0927		149.91	0.0882		152.60	0.0836	
	CA1	_	_		-0.9454	0.3271		-1.14	0.2573	
	CA2	_	_		_	_		0.67	0.4785	
Writing	LOI2009	-39.08	0.0015	1105.7	-39.52	0.0013	1106.2	-39.26	0.0014	1106.7
	Tree cover	-68.47	0.2728		-70.29	0.2593		-74.17	0.2335	
	LOI2009xTree cover	136.86	0.0920		139.47	0.0854		143.91	0.0756	
	CA1	_	_		-1.15	0.1958		-1.47	0.1118	
	CA2	_	_		_	_		1.10	0.2033	
Math	LOI2009	-57.11	0.0005	1187.1	-57.85	0.0004	1186.4	-57.29	0.0004	1183.7
	Tree cover	-153.65	0.0647		-156.79	0.0579		-165.43	0.0430	
	LOI2009xTree cover	250.05	0.0209		254.53	0.0180		264.43	0.0130	
	CA1	_	_		-1.98	0.0932		-2.69	0.0268	
	CA2	_	_		_	_		2.46	0.0310	
Mean of all test results	LOI2009	-46.06	0.0005	1126.7	-46.57	0.0005	1126.7	-46.26	0.0005	1126.7
	Tree cover	-98.93	0.1414		-101.11	0.1315		-105.93	0.1131	
	LOI2009xTree cover	176.32	0.0440		179.43	0.0398		184.96	0.0336	
	CA1	_	_		-1.38	0.1501		-1.77	0.0740	
	CA2	_	_		_	_		1.37	0.1406	

**Fig 3:**
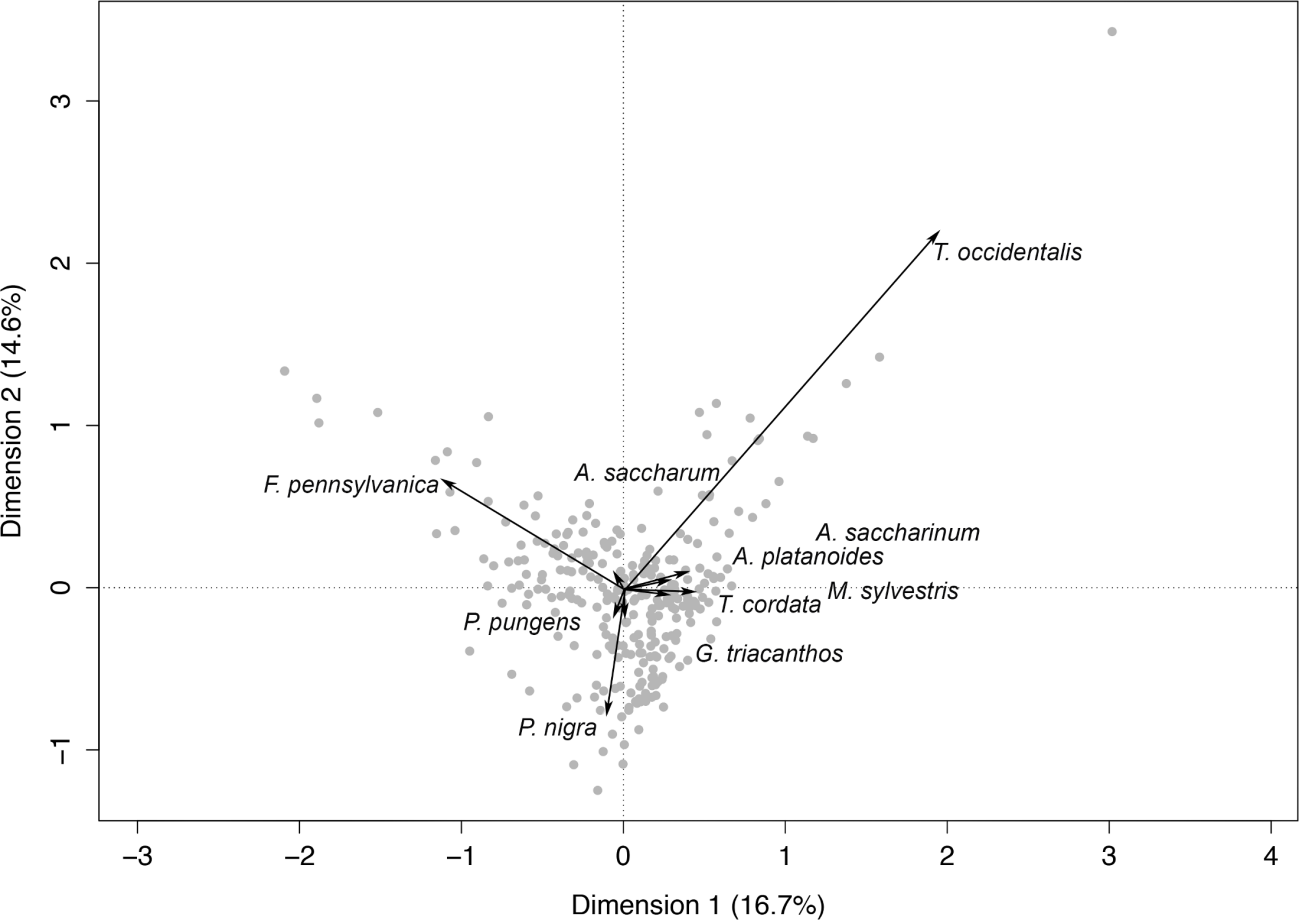
Correspondence analysis of species composition data on axis 1 and 2 from schools (n = 281) within the Toronto District School Board in Toronto, Ontario, Canada. Species axis scores are shown by arrows, and schools by solid circles. The following species are *Thuja occidentalis* (14.2% of the total inertia), *Fraxinus pennsylvanica* (13.6% of the total inertia), *Pinus nigra* (11.2% of the total inertia), *Tilia cordata* (10.7% of the total inertia), *Acer saccharinum* (10.1% of the total inertia), *Picea pungens* (9.3% of the total inertia), *Acer platanoides* (8.3% of the total inertia), *Acer saccharum* (7.7% of the total inertia), *Malus sylvestris* (7.7% of the total inertia), and *Gleditsia triacanthos* (7.1% of the total inertia).

Species composition data were explored in more detail with correspondence analysis (Fig 3). The first two principal axes accounted for 16.7 + 14.6 = 31.3% of the total inertia in the model. The projections of the points onto the first and second axes are shown in Fig 3. *Fraxinus pennsylvanica*, *Pinus nigra*, *Picea pungens*, and *Acer saccharum* were the species positively associated with dimension 1; *Thuja occidentalis*, *Tilia cordata*, *Acer saccharinum*, *Acer platanoides*, *Malus sylvestris*, *Gleditsia triancanthos* were negatively associated. *Thuja occidentalis*, *Fraxinus pennsylvanica*, *Acer saccharum*, *Acer saccharinum*, and *Acer platanoides* were the species most positively associated with axis 2, while *Pinus nigra*, *Gleditsia triancanthos*, *Picea pungens*, *Malus sylvestris*, *Tilia cordata* were negatively associated. All species made a contribution of more than 7% to the total inertia. Correspondence analysis on axes 1 (CA1) and 2 (CA2) were included in GLMs accounting for schools ranked higher on LOI (≥0.5) (Table 2), and they significantly (P = 0.031, P = 0.001) improved math test results (Table 2). The AICc decreased for math test results by 3.4 units. Species composition effects were detectable on math test results (P = 0.03) when CA1 and CA2 were used as predictors. In contrast, species composition effects were absent in reading (P = 0.26, P = 0.48), writing (P = 0.11, P = 0.20) and mean test (P = 0.07, P = 0.14) results for both CA1 and CA2. In all cases, coefficients describing the effects of CA1 were positive while the effects of CA2 were negative.

## Discussion

We found evidence to support our general hypothesis that tree cover has a positive effect on children’s academic performance controlling statistically for the predominant effects of socioeconomic factors. Tree species diversity and relative abundance of conifers did not have detectable effects on children’s academic performance; however, correspondence analysis results showed that species composition did have significant effects. Our results suggest that urban school districts can improve children’s academic performance by increasing tree cover, in particular by focusing on socio-economically disadvantaged schools. We found that socio-economic factors were the most important influence on children’s academic performance, particularly in early school years, consistent with previous studies [17–18, 27]. However, we also found a positive relationship between tree cover and the academic performance of grade 6 children, even after adjusting for LOI. These results support existing work that demonstrates an association between academic performance and “green space” [21–22].

Unlike previous studies that have examined effects of green space (including soft surface and trees) [21–22], the present study highlights tree cover as a more pronounced predictor of children’s academic performance than other vegetation types. Tree cover showed positive correlations with children’s academic performance (Fig 2A and 2D); however, the proportion of soft as opposed to paved surface (Fig 2B) did not have any significant relationship with test results. Similar patterns emerged in analyses examining effects of the proportion soft surface (Fig 2E) on highly challenged schools (LOI ≥5).

Tree species diversity (Fig 2C and 2F) did not have a significant influence on children’s academic performance. This is likely explained in part by a lack of variation in tree diversity found in schoolyards, reducing the statistical power to detect a diversity effect. Although our multiple regression models did not detect tree diversity effects, species composition quantified using correspondence analysis (CA) did have a detectable effect. The first CA axis, which showed a positive effect on test scores, was positively associated with a mix of commonly planted tree species (i.e., *Pinus nigra*, *Picea pungens*, *Acer saccharum*, *and Fraxnius pennsylvanica*). We, therefore, speculate a combination of conifers and hardwoods to have a positive effect on children’s academic performance. Contrary to our hypothesis, the proportion of conifers had no detectable effect independent of total tree cover, but the combination of these observed species had detectable effects on children’s academic performance. Our results thus specifically highlight the importance of planting a diversity of tree species including conifers and deciduous trees in schoolyards.

Statistical effects of species composition were more pronounced for math than for reading or writing components of the EQAO tests. According to cognitive and learning literature, math anxiety is described as the tension and fear that intervenes in one’s ability to manipulate numbers in normal day-to-day life and academic settings [28]. High anxiety disrupts the working memory responsible for completing math-related tasks [29]. Our results are thus consistent with the hypothesis that exposure to a healthy and diverse array of tree species reduces feelings of anxiety allowing for an improved working memory to deal with complicated math problems.

In terms of psychological mechanisms, the observed positive correlations between children’s academic performance and tree cover are consistent with the Attention Restoration Theory that proposes contact with nature restores and redirects one’s attention to the current task at hand [30]. The theory postulates that mental fatigue increases irritation, distraction, and stress and decreases the ability to concentrate. In order to counter the effects of fatigue and restore mental acuity, the theory proposes that clearing the mind, redirecting attention, dealing with unresolved concerns, and reflecting on priorities can all be better achieved in a supportive environment that includes green vegetation [30–33]. Our observed correlations are also consistent with psycho-evolutionary theory, which suggests that natural settings can have a stress-reducing and calming effect on individuals by regulating their emotional responses to their environment. Natural environments can positively impact an overall sense of emotional well-being by lowering neurophysiological stress [32–33], thereby generating more positive emotions, sustaining attention, and restricting negative thoughts. Both theories support the idea that nature functions as a restorative and stress-reducing environment for humans, and our results suggest that tree cover is specifically important in inducing this effect.

Our results may be of use to school boards such as the TDSB to help rationalize an increased expenditure on greening schoolyards, as often these expenditures are considered purely aesthetic and low priority among school board management. Our results specifically point to the importance of increasing tree cover and planting a diverse array of trees on grounds of schools facing external socio-economic challenges. Continued opportunities for outdoor exposure are also likely critical to realize these positive effects (e.g., the Toronto District School Board schools encourages a minimum of 1.5 hours/day of outdoor play time for their primary school children). In addition to potential academic benefits, there is broad evidence that students gain additional benefits from increased tree cover on school grounds, including increased physical activity [13], perceived safety [9], and a variety of other health-related benefits such as attenuation of UV radiation exposure and reduced temperature extremes [2–7]. Planting and maintenance of trees typically comprise less than 0.1% of school board budgets; small investments in this area may result in surprisingly large impacts on learning outcomes.
